# Multi-objective optimization for RNA design with multiple target secondary structures

**DOI:** 10.1186/s12859-015-0706-x

**Published:** 2015-09-03

**Authors:** Akito Taneda

**Affiliations:** Graduate School of Science and Technology, Hirosaki University, 3 Bunkyo-cho, Hirosaki, Aomori, Japan

**Keywords:** RNA switch, Artificial riboswitch, Multi-objective genetic algorithm, Pseudoknot

## Abstract

**Background:**

RNAs are attractive molecules as the biological parts for synthetic biology. In particular, the ability of conformational changes, which can be encoded in designer RNAs, enables us to create multistable molecular switches that function in biological circuits. Although various algorithms for designing such RNA switches have been proposed, the previous algorithms optimize the RNA sequences against the weighted sum of objective functions, where empirical weights among objective functions are used. In addition, an RNA design algorithm for multiple pseudoknot targets is currently not available.

**Results:**

We developed a novel computational tool for automatically designing RNA sequences which fold into multiple target secondary structures. Our algorithm designs RNA sequences based on multi-objective genetic algorithm, by which we can explore the RNA sequences having good objective function values without empirical weight parameters among the objective functions. Our algorithm has great flexibility by virtue of this weight-free nature. We benchmarked our multi-target RNA design algorithm with the datasets of two, three, and four target structures and found that our algorithm shows better or comparable design performances compared with the previous algorithms, RNAdesign and Frnakenstein. In addition to the benchmarks with pseudoknot-free datasets, we benchmarked MODENA with two-target pseudoknot datasets and found that MODENA can design the RNAs which have the target pseudoknotted secondary structures whose free energies are close to the lowest free energy. Moreover, we applied our algorithm to a ribozyme-based ON-switch which takes a ribozyme-inactive secondary structure when the theophylline aptamer structure is assumed.

**Conclusions:**

Currently, MODENA is the only RNA design software which can be applied to multiple pseudoknot targets. Successful design results for the multiple targets and an RNA device indicate usefulness of our multi-objective RNA design algorithm.

**Electronic supplementary material:**

The online version of this article (doi:10.1186/s12859-015-0706-x) contains supplementary material, which is available to authorized users.

## Background

In synthetic biology, biological systems are treated as a circuit composed of biomolecular parts such as nucleic acids and proteins. Since not only natural biomolecules but also artificially-constructed ones can be used as the molecular parts for constructing biological circuits, various efforts have been made to design novel biomolecules which have a desired function. In this context, synthetic RNA devices utilizing a conformational change have intensively been investigated and applied to control biological processes such as gene expression [1–4]. Since we can design various synthetic RNAs by combining the switching ability with a variety of natural RNA functions including enzyme [5], molecular recognition [6], thermometer [7], guide sequence [8], and scaffold [9], artificial RNA sequences with structural changes give a promising platform for creating biomolecular devices which control biological functions in accordance with the designer’s purpose.

We have to take secondary structure into account when designing an artificial RNA whose function needs a specific secondary structure. To date, manual/experimental approaches [1, 10] and computational designs [2–4, 11–14] have been proposed for the rational design of functional RNAs. In case that an automated tool for rational design does not exist for a desired functional RNA, secondary structure prediction method has been utilized in a trial-and-error manner [15]. Since such a trial-and-error approach can be a time-consuming process, a more automated tool for the rational design is important for the efficient development of RNA devices. Biomolecular design algorithms which find a biological sequence folding into a prescribed target structure are called ‘inverse folding’. The inverse folding of RNA can be formulated as a combinatorial optimization problem, in which a discrete space is explored to find an RNA sequence folding into a specified secondary structure [16–23]. The inverse folding algorithms of RNA can be classified into two categories: those for a single target and for multiple targets. Multi-target inverse folding designs the RNA sequences which fold into user-prescribed multiple secondary structures. Since conformational changes can be encoded in such multiple target structures, multi-target inverse folding is particularly useful for designing the RNA sequences with structural changes. For example, synthetic riboswitches and RNA devices are important targets of such designs. So far, the multi-target inverse folding methods have utilized single-objective optimization frameworks, where a weighted sum of objective functions (OFs) is optimized to obtain desired RNA sequences [3, 24–26]. However, the choice of weight parameter values can become rather empirical and can be a tedious task. An *in silico* selection pipeline has also been used to design synthetic riboswitches [4], where multiple criteria are used in a step-by-step manner to filter randomly generated RNA sequences. In the present study, we propose a multi-target inverse folding algorithm for RNA, which is based on multi-objective genetic algorithm (MOGA) [27]. MOGA is a framework suitable for the optimization problem with multiple OFs. Our multi-target design algorithm for RNA has been developed as a new version of our previous single-target RNA design algorithm, MODENA [28], which is based on MOGA. By using the multi-target version of MODENA, we can explore the optimal multistable RNA sequences without empirical weight parameters among multiple OFs. In addition, it is noteworthy that MODENA is the first inverse folding software which can perform the RNA design for multiple pseudoknot targets. In the rest of the present paper, we will describe our algorithm for multi-target RNA design in detail. Then we will show the design performance and usefulness of our algorithm through the benchmarks for multiple target structures and a design example of an RNA device which is taken from recent literature.

## Methods

### Optimization technique

We denote an RNA sequence of length *N* by *S* = *s*_1_..*s*_*i*_..*s*_*N*_, where *s*_*i*_∈{A, C, G, U}. An RNA secondary structure, *θ*, is defined as a set of base pairs, where a base pair is defined as a pair, (*i*, *j*), of nucleotide positions. We consider only canonical (AU, GC) and wobble (GU) base pairs. An RNA sequence which can form a target secondary structure is called a ‘compatible’ RNA sequence [16].

Multi-target RNA design such as RNA device design is an inherently multi-objective problem since not a single but multiple requirements, e.g. a structure stability and a structure similarity with a target structure, can be needed to specify a desired function of RNAs. We define multi-target and multi-objective RNA sequence design problem as follows: finding an RNA sequence with a length of *N* which is compatible with prescribed multiple target secondary structures *θ*_*i*_ (*i*=1,…,*n*_target_) and is Pareto optimal with respect to given OFs *f*_*i*_ (*i*=1,…,*n*_OF_), where the OFs can be a minimum free energy, the energy difference between two secondary structures, or other predicted values. Moreover, formulae include such predicted values can also be used as the OFs.

Usually, there are trade-offs among the OFs of practical multi-objective problems. In such cases, a single optimal solution does not exist and the best solutions we can expect are Pareto optimal solutions [27]. Pareto optimal solutions are a set of solutions which are not dominated by any other solutions, where solution A is said to dominate solution B if $f_{i}^{\mathrm {A}}$ is superior or equal to $f_{i}^{\mathrm {B}}$ for all *i* and a *j* (1≥*j*≥*n*_OF_) which satisfies $f_{j}^{\mathrm {A}} \ne f_{j}^{\mathrm {B}}$ exists. So far, inverse folding methods which do not utilizing multi-objective optimization techniques have solved the RNA sequence design problems by using a weighted sum of OFs. Since the optimal solutions obtained as a result of such a weighted sum of OFs are included in Pareto optimal solutions [27], multi-objective optimization corresponds to simultaneously exploring multiple solutions which can be obtained by optimizing various weighted sums of OFs. By utilizing the framework of multi-objective optimization, we can explore RNA sequences with complex characteristics without tuning empirical weights among OFs.

**Fig. 1:**
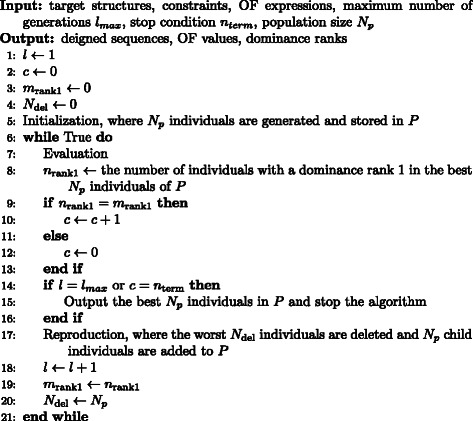
Pseudocode for the genetic algorithm utilized in MODENA for multiple targets *l* is a loop counter; *c* is a counter for a stop condition; *P* is a set of individuals; *n*_rank1_, *m*_rank1_ and *N*_del_ are temporary variables

To explore the optimal RNA sequences for multiple OFs, we use non-dominated sorting genetic algorithm 2 (NSGA2) which is one of the widely used MOGAs [27, 29]. MOGA is a powerful framework for obtaining an approximate set of Pareto optimal solutions and has been applied to various fields of bioinformatics [28, 30, 31]. It is noted that, in the present study, MODENA explores Pareto optimal solutions, whereas the previous versions of MODENA explore weak Pareto optimal solutions [28, 32] (weak Pareto optimal solutions are a set of solutions which are not strongly-dominated by any other solutions, where solution A is said to strongly dominate solution B if $f_{i}^{\mathrm {A}}$ is superior to $f_{i}^{\mathrm {B}}$ for all *i* [27]; we can explore weak Pareto optimal solutions by using option -S in the multi-target version of MODENA). By using user-prescribed parameters (a population size *N*_*p*_, the maximum number of generations *l*_max_, a termination condition *n*_term_, and so on), the multi-target version of MODENA works as follows: (i) Initialization: *N*_*p*_ individuals (RNA sequences) are randomly generated by the nucleotide assignment algorithm (described in a ‘Nucleotide assignment algorithm’ subsection) to fulfill the initial population *P*; (ii) Evaluation: OF values of each individual in *P* are computed, and then a dominance rank and crowding distance [29] are assigned to each individual in *P*; if a stop condition (the number of non-dominated solutions, *n*_rank1_, does not change for a specified number, *n*_term_, of continuous GA generations; in a set of solutions, a solution which is not dominated by any other solution in the set is called a non-dominated solution) is met or the iteration number reaches the maximum number of generations, *l*_max_, stop the algorithm; (iii) Reproduction: *N*_*p*_ child individuals (*C*) are generated by iteratively applying GA operators such as mutation and crossover (described in a ‘GA operators’ subsection) to the parents selected by crowding tournament selection [29] from the best *N*_*p*_ individuals in *P*, where the ‘best’ solutions are defined based on dominance rank and crowding distance [29]; after the generation of the *N*_*p*_ child individuals, we delete the worst individuals from *P* (if necessary) and add *C* to *P* (*P* has 2×*N*_*P*_ individuals here), then go to step (ii) to process the next generation. The pseudocode of the GA utilized in MODENA is described in Fig. 1.

### Objective functions

To design RNA sequences with complex characteristics, it is necessary that the user can utilize various OFs which are a function of predicted sequence properties of the designed RNA, since the design goal of inverse folding can be specified as the maximization/minimization of such OFs. In MODENA algorithm, we invoke the executable files of RNA structure prediction methods, such as RNAfold, for each designed sequence by using a system call and retrieve the predicted results through the standard output or output files. The obtained predicted values (*property* values), e.g. a free energy value, the structure similarity score (= 1−*d*/*N*, where *d* is the structure distance between the target and predicted structures [28]), and the energy barrier height between target structures, are used to evaluate the OFs of each designed RNA sequence. The *methods* and their *properties* which can be used to construct OFs in MODENA are summarized in Table 1. Before running MODENA, the *methods* which have the *properties* to be used to construct OFs are specified as a *method* list by the user. All *properties* of the *methods* specified in the *method* list are computed for each individual at the GA evaluation step. As a result, the computed *property* values are assigned to each individual. For some *methods* (e.g. those with SIM and FE *properties* in Table 1), we have to specify one of the target structures in the *method* list.

**Table 1:** *Methods* and their *properties* available in MODENA. *Method* names are mainly taken from those of corresponding structure prediction methods

Method	Property	Str.	Package
RNAfold	MFE,SIM	y	The Vienna RNA Package [16, 41]
RNAfold-p^a^	MFE,EFE,PB,SIM	y	The Vienna RNA Package [16, 41]
RNAeval	FE	-	The Vienna RNA Package [16, 41]
FindPath^b^	BAR	-	The Vienna RNA Package [16, 41]
Fold	MFE,SIM	y	RNAstructure [42]
fold^c^	FE	-	RNAstructure [42]
EnsembleEnergy	EFE	-	RNAstructure [42]
CentroidFold	FE^d^,SIM	y	www.ncRNA.org [43, 44]
centroidfold^c^	FE	-	www.ncRNA.org [43, 44]
IPknot^e^	GCPAIR,SIM	y	www.ncRNA.org [44, 45]
mfe^e^	MFE,SIM	y	NUPACK [35]
pfunc^e^	EFE,PF	-	NUPACK [35]
energy^e^	FE	-	NUPACK [35]
prob^e^	PB	-	NUPACK [35]
defect^e^	DEF,NDEF	-	NUPACK [35]
UNAFold	MFE,SIM	y	UNAFold [46]
pknotsRG^e^	MFE,SIM	y	RNA studio [47]
HotKnots^e^	MFE,SIM	y	RNAsoft [48]
GC^e^	CONT	-	-

MFE: minimum free energy; SIM: structure similarity between target and predicted secondary structures [28]; EFE: ensemble free energy; PB: Boltzmann probability; FE: free energy of a specified target structure; BAR: energy barrier height between two specified target structures; GCPAIR: GC content of base paired nucleotides multiplied by -1; PF: partition function; DEF: ensemble defect; NDEF: normalized ensemble defect; CONT: GC content. The str. column indicates whether the *method* assigns a predicted structure to each individual or not

^a^RNAfold with option -p

^b^We use accessFindPath.py [34] for the findpath.c [24] in the Vienna RNA package

^c^The secondary structure of all nucleotide positions is constrained to obtain the free energy of a specified secondary structure like RNAeval

^d^Free energy value output by CentroidFold

^e^The *methods* which can deal with pseudoknots

In addition to the structure prediction programs, GC content is also included in the available *properties*. Since biased GC content can easily appear in the designed sequences if we do not take GC content into account [22], |*r*(GC:CONT)−*ρ*_target_| was used in one of the OFs in the present study, where *r*(*X*:*Y*) indicates the value for the *property* *Y* of *method* *X*, and *ρ*_target_ is a user-specified target GC content. The *r*(GC:CONT), or a GC content (%), is calculated by counting the number of Gs and Cs in the designed sequence and dividing the count by the nucleotide length of the sequence.

In MODENA, target GC content can be taken into account through an OF. For this reason, the constraint for GC content is not exact but an approximate one. Since there can be a trade-off between a GC content and another OF value, e.g. a minimum free energy, an OF including a GC content can interfere with another OF during the design.

It is noted that increasing the number of OFs usually makes the design more difficult. In the present study, at most we used five OFs (see the ‘An example of RNA device design’ subsection).

### Sequence and structure constraints

To fix functional motifs during the sequence design process, the sequence constraints in the IUPAC nucleotide code are available in MODENA. In addition to the sequence constraints, we can specify secondary structure constraints for each secondary structure prediction method if the prediction method can use secondary structure constraints (e.g. the Fold program of RNAstructure, CentroidFold, and RNAfold provide such a function). While the constraint sequences are never changed during a design run, the structure constraints are applied only when the prediction method with the structure constraints is invoked, so that we can define and use different structure constraints for each *method*. A typical usage of the structure constraints is modelling of the ligand-binding state of an aptamer. If a ligand exists, the aptamer domain binds the ligand and forms a characteristic secondary structure. In inverse folding, this ligand-binding state can be modelled by using the structure constraints which specify the characteristic secondary structure of the aptamer domain [4]. Such a structure-constraint secondary structure prediction gives the secondary structure which has the lowest energy in the set of all the secondary structures with the constraint secondary structure. This corresponds to the lowest energy structure of when the ligand binds to the aptamer.

### Nucleotide assignment algorithm

In the GA initialization and reproduction steps of the inverse folding, we generate RNA sequences which are compatible with the prescribed target structures. The generation of compatible random sequences in single-target inverse folding is easy even when sequence constraint is imposed on. In the case of the RNA sequence design with multiple targets, however, the more complex base-complementarity relationship among nucleotide positions, called the dependency graph [24, 26], has to be taken into account, since a nucleotide position which forms base-pairs with multiple other positions corresponds to a vertex with a degree > 1 and such nucleotide positions cause a network-like relationship. In MODENA, we do not use the ear decomposition of RNAdesign [26] which is a sophisticated graph coloring algorithm and guarantees uniform sampling of RNA sequences compatible with the target structures. Instead, to generate RNA sequences, we use a naive ‘nucleotide assignment algorithm’ described below.

A dependency graph *G*=(*V*,*E*) is the graph composed of vertices, *V*={1,…,*N*}, representing nucleotide positions and edges, $E=\cup _{i=1}^{n_{\text {target}}} \theta _{i}$, corresponding to the base pairs in target structures. In the two-target problem, each connected component *c*_*i*_ (*i*=1,…,*n*_c_) in the dependency graph belongs to one of isolated vertex, path, and cycle [24]. In addition, more complex graph structures can appear in the dependency graph of the multi-target inverse folding with *n*_target_≥3 [26]. To generate RNA sequences compatible with all the target structures, we have to find a nucleotide code assignment to *V*, by which all base paring relationships specified by *E* are satisfied. As described in the generalized intersection theorem [26], if *G* is bipartite, at least one nucleotide code assignment compatible with *G* exists; if *G* is not bipartite, we cannot assign compatible nucleotide codes to *G* since the bipartiteness is also a necessary condition for the latter.

**Fig. 2:**
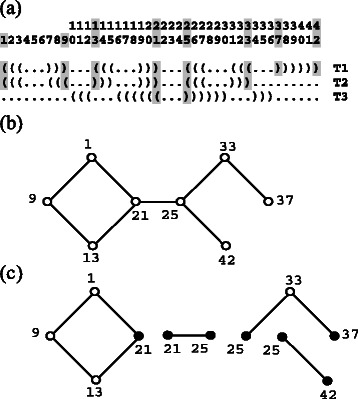
An example of a connected component of the dependency graph. **a** An example of the set of three targets T1, T2, and T3. Nucleotide positions are numbered above the example, e.g, shadowed columns from the left to the right correspond to nucleotide positions 1, 9, 13, 21, 25, 33, 37, 42, respectively. As an example, base paired positions belonging to a connected component are shadowed. The dependency graph is composed of all connected components (not shown except the shadowed one) derived from the targets. **b** A graph representation of the connected component shadowed in (**a**). The numbers indicate nucleotide positions. An edge corresponds to a base pair between nucleotide positions. If there is a base pair in one of the target structures, an edge appears in a connected component. E.g., nucleotide positions 21 and 25 in **a** have a base pair in T3, so that there is an edge between node 21 and 25 in (**b**). **c** Decomposition of the connected component into the paths, where a cycle is treated as a path by defining a single vertex as a ‘start and end’ vertex (e.g. the cycle shown at the left of this figure). The start and end vertices are denoted by solid circles

Since nucleotides can independently be assigned to each connected component, let us consider assigning nucleotides to a connected component *c*. If *c* is an isolated vertex (i.e. the number of vertices |*V*_*c*_| = 1), we assign a nucleotide to the position randomly. When |*V*_*c*_|>1, first we find all the vertices with degree ≠ 2. Any connected component (except for the cases of |*V*_*c*_|=1 and a cycle) can be decomposed into paths which have start and end vertices with degree ≠ 2 (an example is shown in Fig. 2), where each start or end vertex with degree > 2 is shared by multiple paths. If *c* is a cycle, we cannot find the vertex with degree ≠ 2, therefore we arbitrarily select a vertex in the cycle as a ‘start and end’ one.

After the decomposition of the connected component *c* into paths, we enumerate the combinations of compatible nucleotide assignments for each path. The number of the combinations is computed in accordance with the following recursion:

(1) $$\begin{array}{@{}rcl@{}} \chi(k,\mathrm{A},s) &=& \chi(k-1,\mathrm{U},s),  \\ \chi(k,\mathrm{C},s) &=& \chi(k-1,\mathrm{G},s),  \\ \chi(k,\mathrm{G},s) &=& \chi(k-1,\mathrm{C},s) + \chi(k-1,\mathrm{U},s),  \\ \chi(k,\mathrm{U},s) &=& \chi(k-1,\mathrm{A},s) + \chi(k-1,\mathrm{G},s), \end{array} $$

where *k* indicates the position along the path, i.e. *k*=1 corresponds to the start vertex and the *k* for the end vertex is equal to the length of the path; *s* indicates a nucleotide assigned to the start vertex (*s*∈{A, C, G, U}); *χ*(1,*s*,*s*)=1 if a nucleotide *s* is allowed at the start vertex, otherwise 0. A *χ*(*k*,*x*,*y*) gives the number of combinations such that a nucleotide *x* is assigned to the *k*-th vertex in the path when a nucleotide *y* is assigned to the start vertex. For example, if a U and A are assigned to the start and end vertices of path *m*, respectively, and such assignments are compatible for the path, *χ*(*L*_*m*_,*A*,*U*) becomes larger than 0, where *L*_*m*_ is the length of path *m*. It is noted that if a nucleotide *x* is not allowed at the *k*-th vertex due to a sequence constraint, *χ*(*k*,*x*,*s*) is set to 0 during the computation of the recursion.

**Fig. 3:**
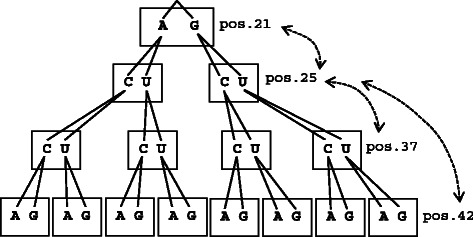
An example of the procedure for assigning nucleotide codes to the ‘start and end’ vertices of the connected component shown in Fig. 2. In this example, position 21 is selected as a root vertex *v*_arb_. Then we consider the tree structure for assigning nucleotides to the ‘start and end’ vertices, where the order of the ‘start and end’ vertices is obtained by the depth-first search in the spanning tree of the connected component (in this example, the order of positions 21, 25, 37, and 42 from the top to the bottom of the tree). Dashed arrows mean that there exists a decomposed path between two positions. In this example, first, an A or G is randomly selected for position 21 (it is noted that, in the case of the nucleotide assignment for the GA initialization, not an A or G, but an A, C, G or U is randomly selected here). Then nucleotide codes of the remaining positions are assigned from the top of the tree to the bottom. Let us consider an A is selected for position 21. Even in the case such that a C alone is allowed to position 37 due to a sequence constraint, first we try to assign AUU from the top to the bottom; however, since this violates the constraint, we backtrack to position 25 and then assign a C to position 37. As a result, we assign AUCA to the ‘start and end’ vertices in this example

From the *χ*(*L*_*m*_,*x*,*y*), we can obtain an indicator function, *λ*(*t*,*u*,*i*,*j*), which gives one if the assignment (nucleotides *t* and *u* are assigned to positions *i* and *j*, respectively) is compatible with respect to the path(s) between positions *i* and *j* (i.e. there exists at least one compatible assignment for each of the paths between positions *i* and *j*), otherwise gives zero. By using the *λ*(*t*,*u*,*i*,*j*), we can assign nucleotides, which are compatible to the target structures, to the ‘start and end’ vertices. First, we assign a nucleotide to the root vertex *v*_arb_ arbitrarily selected from all the ‘start and end’ vertices in the connected component *c*. Then we traverse the ‘start and end’ vertices to assign compatible nucleotides to the rest of the ‘start and end’ vertices (an example is shown in Fig. 3), where the compatibility is checked by using the *λ*(*t*,*u*,*i*,*j*) during the traversal; if we meet a situation such that a compatible assignment does not exist, we backtrack and try a different assignment. During this nucleotide assignment, we give priority to G and C if the *v*_arb_ has a G or C; otherwise, priority is given to A and U. We use this ‘biased’ assignment to reduce the number of GU base pairs in the connected component, while a GU base pair is assigned if a sequence constraint enforces such assignment. After completion of the assignment to all the ‘start and end’ vertices, we assign nucleotides to the vertices with degree = 2 (i.e. the vertices other than the start and end vertices in each path). This assignment can be performed from the end vertex to the start vertex along each path, where a nucleotide at the *k*-th vertex is selected from {*x*|*χ*(*k*,*x*,*s*)>0,*x*∈{A,C,G,U}}, where *s* is a nucleotide assigned to the start vertex of the path. During this assignment also, we give priority to GC or AU base pair over GU base pair.

If a sequence constraint does not exist in the connected component, the nucleotide assignment algorithm uniformly samples the nucleotides assuming AU or GC base pair alone. It is noted that, when a sequence constraint exists, there is no guarantee that the nucleotide assignment algorithm can sample the whole sequence space of a given nucleotide length since we use the biased sampling method in MODENA.

**Fig. 4:**
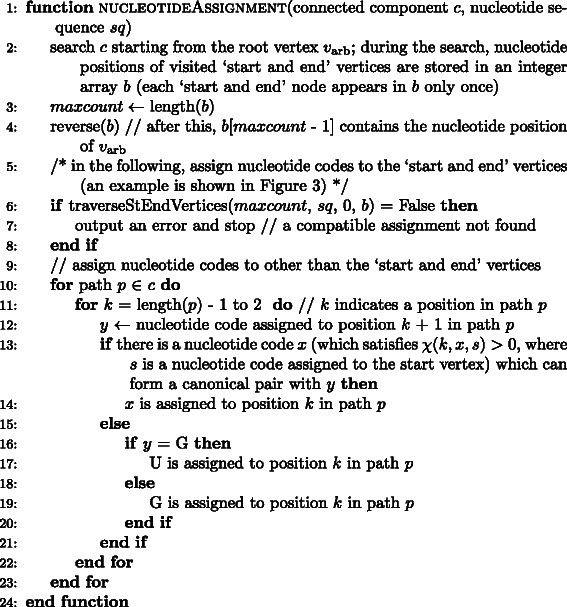
Pseudocode of the nucleotide assignment algorithm for a given connected component *c*. By using this function, we can assign compatible nucleotide codes to the nucleotide positions belonging to *c*; the output is contained in a one-dimensional array *sq*. *maxcount* and *b* are a counter variable and a temporary one dimensional array for traversing the ‘start and end’ vertices, respectively. In line 6, this function calls traverseStEndVertices() shown in Fig. 5. In line 11, *k* = 1 and *k* = length(*p*) correspond to the start and end vertices, respectively; length(*p*) is the number of nucleotide positions (including the start and end vertices) of which path *p* is comprised. In line 13, we assume that *χ*(*k*,*x*,*s*) can be accessed as a global array. Array indices start at 0. Comments are written in the C language-like format

**Fig. 5:**
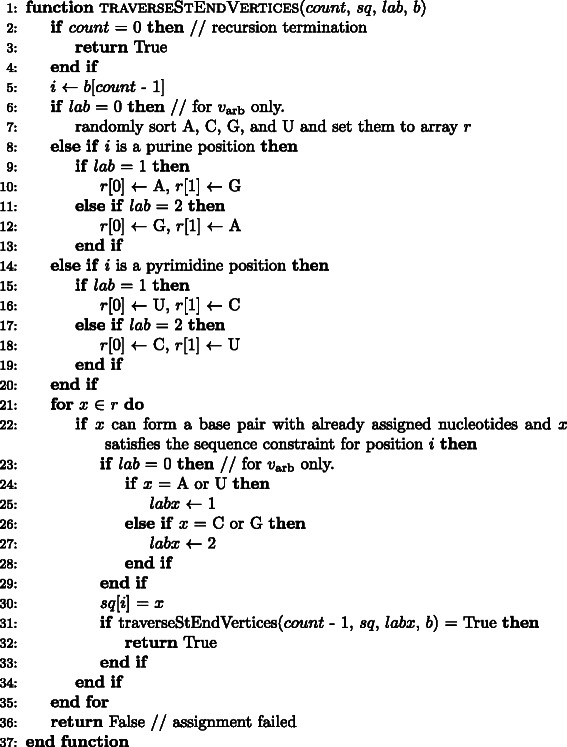
Pseudocode of the nucleotide assignment algorithm (continued from Fig. 4). *i* is a nucleotide position. *sq* is a one-dimensional array for containing a nucleotide sequence. *b* is a one-dimensional array containing nucleotide positions of a connected component. *lab* = 0, *lab* = 1, and *lab* = 2 indicate “nucleotide position *i* is *v*_arb_”, “A and U have a priority”, and “C and G have a priority”, respectively. *x* and *labx* are temporary variables. In lines 8 and 14, a nucleotide type (i.e. purine or pyrimidine) can be determined based on the nucleotide type of *v*_arb_ (which is assigned in line 23 - 29) and the partition, to which position *i* belongs, of the bipartite graph of the connected component *c*. In line 22, we assume that the indicator function *λ*(*t*,*u*,*i*,*j*) is used to check the compatibility as a global array. In line 31, this function calls itself recursively. By resricting possible nucleotide types to purine or pyrimidine in line 7, this function becomes that for transversion operator. Array indices start at 0. Comments are written in the C language-like format

Pseudocode for the nucleotide assignment algorithm is shown in Figs. 4 and 5.

### GA operators

In the reproduction step of GA, individuals in a population are modified by using ‘GA operators’ to generate the next GA population. The multi-target version of MODENA uses four new GA operators: point mutation, negative design, positive design, and crossover operators. Whereas the point mutation and crossover operators are straight forward extensions of the previous GA operators used in the single-target version of MODENA [28], positive and negative design operators are newly introduced operators in the multi-target version. The details of the four GA operators are described below.

#### Point mutation for multiple targets

Mutation is one of the most fundamental operations in heuristic optimization algorithms, since it corresponds to a local move in a search space. In the point mutation, we scan the RNA sequence from the 5’ side to the 3’ side to randomly select a nucleotide position, *i*_mut_, in accordance with a mutation probability *p*_*M*_ (a default value *p*_*M*_ = 0.05); at each nucleotide position, we generate a random number, *r*_n_, where 0.0 ≤*r*_n_<1.0, and compare *r*_n_ and *p*_*M*_; if *r*_n_≤*p*_*M*_, we try to mutate the nucleotide position, otherwise we do nothing and move on to the next nucleotide position. If the selected position belongs to a loop nucleotide, the selected nucleotide is simply changed to a nucleotide different from the original one with an equal probability; if the selected position forms a base pair in the target secondary structures, we randomly select and apply one of transversion and transition operators to the selected nucleotide. If the transversion or transition operator failed (this can occur due to a sequence constraint), the nucleotides of the connected component including the selected nucleotide are not changed.

If we introduce a transversion at a nucleotide position belonging to a connected component, all the other positions in the connected component must change their nucleotides to repair the compatibility, since any connected component is bipartite and all nucleotide positions belonging to each partition must have the same nucleotide type (purine or pyrimidine). This repair process is performed in accordance with the ‘nucleotide assignment algorithm’.

In the case of transition operator, there exists a case where one nucleotide change (i.e. a change in the randomly selected position) alone is adequate. For example, the A of an AU base pair can be changed to a G, leading to a GU base pair and this change does not destroy the compatibility of the original base pair. In other cases, however, more nucleotide changes can be required to guarantee the compatibility. Therefore, we traverse the spanning tree of the connected component in order of the breadth-first search to repair the compatibility, where the vertex of nucleotide position *i*_mut_ is the root vertex of the spanning tree. Similar to the case of two-target inverse folding [24], the traversal up to depth one is adequate to repair any connected component of multiple targets.

In the point mutation, one of the transversion and transition operators is randomly selected with the equal probability (= 0.5) and applied to each nucleotide position selected with the *p*_*M*_.

#### Negative design operator

**Fig. 6:**
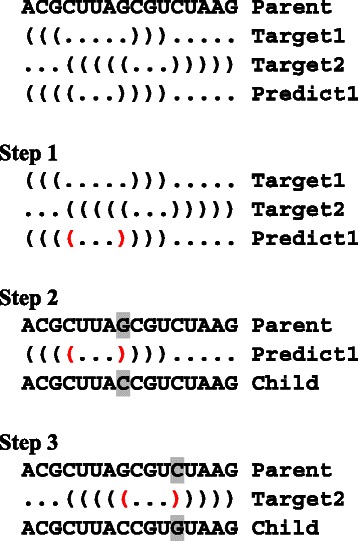
An example of negative design operator. Here we consider a two-target design. In this example, a parent sequence, target structures 1 and 2, a selected predicted structure, and a child sequence are denoted by Parent, Target1, Target2, Predict1, Child, respectively. First, the Parent sequence is copied to the Child sequence; Step 1) we detect undesired base pairs (positions are denoted in red) in Predict1 which do not appear in the target structures; Step 2) we change the nucleotide(s) of each undesired predicted base pair to disrupt the undesired base pairing. Here, the G in Parent is changed to the C in Child (both are denoted by shadowed characters); Step 3) to repair the base complementarity denoted in red, the shadowed C is changed to the shadowed G

To accelerate the convergence of the GA optimization process, we use a GA operator, negative design operator, with greedy nature. RNA negative design means reducing undesired base-pairing and its notion has been exploited to design structured RNA sequences, e.g. in the context of the Newtonian dynamics model [23] and GA [28]. In our negative design operator, first we have to detect undesired base pairs which do not appear in the target structures. Here, we focus on the undesired base pairs appear in a predicted secondary structure. If the parent individual has multiple predicted secondary structures (this corresponds to the case in which multiple structure prediction methods are listed in the *method* list), the *method* which has the lowest structure similarity score (which is computed as a *property* for a specified target structure) is selected from the *method* list and its predicted secondary structure is used. After comparison between each of all target structures and the selected predicted structure, we change the nucleotide(s) of each undesired predicted base pair to disrupt the undesired base pairing; if the connected components of both nucleotides are isolated vertices with |*V*_*c*_|=1, both nucleotides can be changed; otherwise, one of the two nucleotides is changed. After this step, repair of the connected component containing the modified nucleotide is performed by using transversion or transition operator, if necessary. In negative design operator, we scan the RNA sequence from the 5’ side to the 3’ side to try to disrupt undesired base pairs. We do not change any nucleotide of the undesired base pairs if both nucleotides of the undesired base pair belong to the same connected component. An example of negative design operator is shown in Fig. 6.

#### Positive design operator

**Fig. 7:**
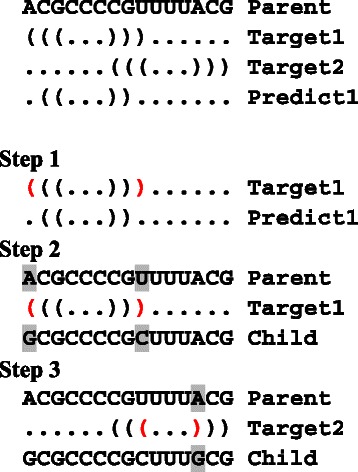
An example of positive design operator. Here we consider a two-target design. In this example, a parent sequence, target structures 1 and 2, a selected predicted structure, and a child sequence are denoted by Parent, Target1, Target2, Predict1, Child, respectively. First, the Parent sequence is copied to the Child sequence; Step 1) we scan Predict1 from the left to the right to find a target base pair which is missing in Predict1 (denoted by a red base pair); Step 2) we change the shadowed A in Parent to the shadowed G in Child. Then, to repair the base complementarity, the shadowed U is changed to the shadowed C; Step 3) in addition, since the changed position forms a base pair in Target2, the shadowed A is changed to the shadowed G

This operator is another greedy GA operator introduced in the multi-target version of MODENA. While the negative design operator tries to eliminate undesired base pairs, this operator has a role to make desirable base pairs more stable if possible. Positive design operator scans the RNA sequence from the 5’ side to the 3’ side to assign a GC base pair to the nucleotide positions which form a base pair in a selected target structure but do not form a base pair in a predicted structure. The target and predicted structures are selected in the way similar to the case of the negative design operator (i.e. we select the *method* having the lowest structure similarity score and its target and predicted structures are used as the selected target and predicted structures). To the upstream nucleotide position, *i*_up_, of a target base pair which is missing in the selected predicted structure, positive design operator assigns a G or C in accordance with whether the position is a purine or pyrimidine position. After that, we repair the rest of the connected component (to which the upstream nucleotide position belongs) by a ‘GC-biased’ transition operator. The ‘GC-biased’ transition operator gives priority to a G and C during the nucleotide assignment. To increase the number of GC pairs, this operator can change the nucleotides at a depth deeper than one in the spanning tree of the connected component, where the vertex of nucleotide position *i*_up_ is the root vertex of the spanning tree (e.g. if a U of a connected component composed of only AU base pairs is changed to C, this operator can change nucleotides located at a depth deeper than one in the spanning tree to increase GC pairs). To maintain the local-move nature of this operator, this traversal is restricted to a prescribed maximum depth (a default value = 3) of the spanning tree. An example of positive design operator is shown in Fig. 7.

Since positive design operator tries to increase the GC content of the base-paired positions in the target structures, positive design operator may cause a slow convergence in the GA when the user designs the RNA sequences with a low GC content. Positive design operator can be turned off by option “-opPos 0”.

#### Crossover for multiple targets

Crossover operator combines subsequences taken from two ‘parent’ individuals to generate a new one and can give a ‘long jump’ in the search space in contrast to the local move of the mutation operator. Let us consider two parent individuals (called parent L and R). First, a nucleotide position (a crossover point) *p* is selected at random (1≤*p*≤*N*−1), and then we split each parent individual into the 5’ half (*s*_1_..*s*_*p*_) and the 3’ half (*s*_*p*+1_..*s*_*N*_) of the original sequence at the selected nucleotide position. Then, we try to splice the 5’ half of parent L and the 3’ half of parent R to generate a child individual. If there exists a connected component whose nucleotides distribute both in the 5’ half and 3’ half, the nucleotides belonging to the connected component are copied from one of the parents to the child (in the current version of MODENA, those of parent L are copied).

### Mutation of undesired sequence motifs

To avoid undesired functions, we implemented simple operators for mutating nucleotide tracts and user-prescribed sequence motifs. Switching on/off of these operators can be specified through options. These operators are invoked just after (i) the processing by each GA operator and (ii) the GA initialization of each individual. If a nucleotide tract or specified sequence motif is found in the designed sequence, the point mutation is performed at a nucleotide position in the detected region to change the sequence.

## Results and discussion

To show the optimization performance of the multi-target version of MODENA, we performed computational RNA design for the various sets of multiple targets and sequence constraints. Throughout the rest of the present paper, we use a population size of 100, the maximum number of generations of 200, and target GC content *ρ*_target_ = 50 (%) for MODENA if the other values are not mentioned. For performance comparison, we ran RNAdesign with option “-n 500 –thin 200 -b 100 –scale 1” and extracted top 100 RNA sequences as the results of RNAdesign; we used Frnakenstein with option “-s 100” to design 100 RNA sequences. When the Vienna RNA Package is required in the present study (for sequence design and performance evaluation), MODENA and Frnakenstein used Vienna RNA Package 1.8.3, and RNAdesign used Vienna RNA Package 2.1.1. NUPACK 3.0 and RNAstructure 5.3 were also used for pseudoknotted RNA design and RNA device design by MODENA, respectively.

### Two-target design

As design examples of the two-target inverse folding without sequence constraint, we designed RNA sequences which fold into the metastable structures of SV11 [33], the 17 sets of metastable structures taken from the dataset (command_linesNew.faa) used in the paper of RNAtabupath [34], and two-target pseudoknot datasets (datasets generated by NUPACK:subopt [35] and that based on the natural pseudoknots taken from Pseudobase [36]). For the pseudoknot-free two-target designs, we minimized the following three objective functions computed by RNAfold and RNAeval with option -d2: $f_{1}=\sum _{i}^{n_{\text {target}}}(E(\theta _{i})-G)$, $f_{2}=\sum _{i < j}|E(\theta _{i})-E(\theta _{j})|,f_{3}=|r(\text {GC:CONT})-\rho _{\text {target}}|$, where *G* and *E*(*θ*_*i*_) are the ensemble free energy and the free energy of target structure *θ*_*i*_, respectively. In the multi-target pseudoknot designs of the present study, instead of RNAfold and RNAeval, we used the ‘pfunc’ and ‘energy’ of NUPACK with option -pseudo to evaluate *f*_1_, *f*_2_, and *f*_3_.

SV11 is the RNA molecule frequently used to test the design performance of the multi-target inverse folding methods [24–26]. As shown in Table 2, MODENA successfully designed the RNA sequences with the metastable SV11 structures. It is noteworthy that we successfully designed 19 ‘completely multistable’ RNA sequences in which both of the SV11 metastable structures have the lowest free energy. As the other two methods failed to obtain the completely multistable structures, achieving the completely multistable design in this design problem is not trivial.

**Table 2:** The results of two-target designs

		MODENA				RNAdesign				Frnakenstein			
RNA	*l*	*δ* *e*_1_	*δ* *e*_2_	*n*_1_	*n*_2_	*δ* *e*_1_	*δ* *e*_2_	*n*_1_	*n*_2_	*δ* *e*_1_	*δ* *e*_2_	*n*_1_	*n*_2_
SV11	115	0.00	0.00	100	19	0.00	0.20	11	0	0.00	0.50	84	0
alpha operon	130	0.10	0.60	0	0	0.10	0.40	3	0	0.00	0.60	34	0
amv	145	0.60	0.70	0	0	1.30	2.20	0	0	0.80	2.40	1	0
attenuator	73	0.00	0.80	33	0	3.50	3.80	0	0	0.30	0.60	0	0
dsrA	85	0.00	0.00	100	7	0.00	0.10	15	0	0.00	0.80	99	0
HDV	153	0.00	0.00	100	30	0.00	0.10	11	0	0.00	0.50	100	0
HIV-1 leader	280	0.50	0.70	0	0	1.20	1.50	0	0	0.50	1.30	0	0
ms2	73	0.40	0.40	44	0	1.70	1.80	0	0	0.20	0.40	0	0
rb1	148	0.00	0.04	75	0	0.20	0.40	1	0	0.00	0.66	100	0
rb2	113	0.00	0.00	100	89	0.10	0.20	8	0	0.00	0.10	93	0
rb3	141	0.10	0.10	52	0	1.00	1.50	0	0	0.00	0.00	39	2
rb4	146	2.10	5.60	0	0	3.30	3.70	0	0	3.21	5.31	0	0
rb5	201	0.46	0.50	0	0	0.80	1.30	0	0	0.20	0.44	0	0
ribD leader	304	0.80	1.20	0	0	3.90	4.20	0	0	0.10	2.60	0	0
s-box leader	247	0.70	0.80	0	0	1.60	1.80	0	0	0.30	1.10	0	0
s15	74	0.10	0.40	0	0	0.00	0.40	15	0	0.00	0.20	98	0
SL	56	0.00	0.00	100	28	0.00	0.00	17	1	0.00	0.00	86	6
thiM leader	165	0.60	1.00	0	0	2.70	3.60	0	0	0.36	1.56	0	0
mean	-	0.38	0.76	-	-	1.26	1.58	-	-	0.35	1.09	-	-
median	-	0.10	0.50	-	-	1.00	1.50	-	-	0.10	0.60	-	-

*l* is the nucleotide length of the target set. *δ* *e*_1_ and *δ* *e*_2_ columns show the energy difference between the ground state and the lowest energy target structure and the difference between the ground state and the highest energy target structure, respectively. The values of *δ* *e*_1_ and *δ* *e*_2_ are those for the designed sequence with the lowest *δ* *e*_2_ in the 100 designed sequences, where the RNA sequence with the lowest *δ* *e*_1_ was selected if multiple RNA sequences have the lowest value of *δ* *e*_2_. The *n*_1_ is the number of the designed sequences such that one of the two target structures has the lowest free energy. The *n*_2_ is the number of the designed sequences in which both target structures have the lowest free energy. The target structures of SV11 are taken from the dataset of RNAdesign [26]. We use the RNA names consistent with the dataset of RNAtabupath (Table 1 of [34]). The target structures of the seventeen sets (from alpha operon to thiM leader) are taken from the dataset of RNAtabupath [34] which contains the dataset of paRNAss [49]; the metastable structures of rb1, rb2, rb3, rb4 [50], and rb5 [51] are taken from the literature. The means and medians are those for the seventeen sets of target structures (the result of SV11 is not used to calculate the means and medians)

The design results for the 17 sets of two target structures are also tabulated in Table 2. As shown in the *n*_1_ column of Table 2, for the eight sets of the 17 sets, MODENA designed the RNA sequences such that at least one of the two target structures has the lowest free energy. RNAdesign designed a less number (seven sets of the 17 sets) of such RNA sequences; Frnakenstein did nine sets of the 17 sets, which are slightly better results compared to those obtained by MODENA. Moreover, as can be seen in the *n*_2_ column of Table 2, MODENA successfully designed, for four sets of the 17 sets, the RNA sequences in which both target structures have the lowest free energy, while RNAdesign and Frnakenstein obtained such completely multistable RNA sequences for only one and two sets of the 17 sets, respectively. These results indicate that MODENA has a better design ability for the two-target inverse folding of RNA compared with RNAdesign, and has a design ability comparable to Frnakenstein.

We performed Wilcoxon’s signed rank tests by using R version 3.0.3 for the *δ* *e*_1_ and *δ* *e*_2_ in Table 2, where *δ* *e*_1_ is the energy difference between the ground state and the lowest energy target structure (*δ* *e*_2_ is that for the highest energy target structure). As a result of the comparison between MODENA and RNAdesign, we obtained *P* = 7.324×10^−4^ and *P* = 7.996×10^−3^ for *δ* *e*_1_ and *δ* *e*_2_, respectively. When we compared the design results of MODENA and Frnakenstein, the *P*s for *δ* *e*_1_ and *δ* *e*_2_ were 0.4639 and 3.076×10^−2^, respectively. If we use the Bonferroni correction for these four tests, the *P*s for the comparisons between MODENA and RNAdesign are statistically significant (< 0.05 /4). Since we tested four comparisons (a comparison between *δ* *e*_1_s of MODENA and RNAdesign, *δ* *e*_2_s of MODENA and RNAdesign, *δ* *e*_1_s of MODENA and Frnakenstein, and *δ* *e*_2_s of MODENA and Frnakenstein),“0.05 divided by four” was used here as the level of statistical significance for each comparison.

At the bottom of Table 2, the means and medians for *δ* *e*_1_ and *δ* *e*_2_ are also shown. Better values of the means and medians for genetic algorithms (MODENA and Frnakenstein) imply that better optimization techniques are effective for these design problems. A representative designed sequence for each design problem is tabulated in Additional file 1: Table S1.

As utilized in the computational design of ribozymes by Dotu et al. [14], the Boltzmann probability of each target structure can be a useful measure for evaluating designed RNAs. In the paper by Dotu et al. [14], the designed RNA sequences are classified in accordance with whether the single target structure has a Boltzmann probability ≥ 0.4 or not. In the case of multistable RNA design, it is desirable that all target structures have the same large probability. The Boltzmann probabilities of the RNA sequences designed for the 17 sets of two targets are shown in Additional file 3: Figure S1. As can be seen from the figure, MODENA successfully designed the RNA sequences with (the sum of the Boltzmann probabilities of two target structures) ≥ 0.4 for four target sets (dsrA, HDV, rb2, and SL), while Frnakenstein and RNAdesign designed such RNA sequences only for one (SL) and no set, respectively.

#### Two-target pseudoknot design

MODENA can design pseudoknotted RNA sequences if the structure prediction *method* can predict a pseudoknot [32]. For this reason, the pseudoknot classes which can be designed by MODENA are dependent on those of the structure prediction method. To our knowledge, this is the first report on the inverse folding algorithm for multiple target pseudoknots. As the feasible design problems for the design performance test, by using NUPACK:subopt, we generated 50 and 30 sets of two target structures with a length of 60 and 80 nucleotides, respectively. Hereafter, we call the 50 and 30 sets as PK60 and PK80 dataset, respectively. The PK60 and PK80 datasets were constructed as follows. First we randomly generate an RNA sequence, then perform NUPACK:subopt to obtain two suboptimal structures. We filter the set of two structures by examining the following criteria. (i) (the Hamming distance between the two structures in bracket notation)/(target structure length) ≥ 0.1 to avoid very similar structures. (ii) (the number of base-paired nucleotides of the two structures)/(the sum of the lengths of the two structures) ≥ 0.2 to avoid too many loop nucleotides in the target structures. (iii) At least one pseudoknot is included in one of the two structures. If the set of two structures satisfied these criteria, we added the set to our dataset, otherwise rejected the set. We continued this target set generation process until 50 or 30 sets of two structures were obtained for the PK60 and PK80 datasets, respectively.

In these designs of the pseudoknotted RNAs with multiple targets, we used smaller parameter values, a population size of 30 and the maximum number of generations of 50, to limit the computational times since the pseudoknot prediction can take a very long computational time. In these design benchmarks, MODENA successfully designed completely multistable RNA sequences for a number of the sets of target structures; for the PK60 dataset, we obtained 45 sets (90 % of the 50 sets) in which at least one target structure has the lowest free energy and obtained 27 sets (54 %) which have a completely multistable RNA sequence; in the PK80 dataset, we successfully designed 27 sets (90 % of the 30 sets) in which one of the two targets has the lowest free energy and the 15 sets (50 %) having a completely multistable RNA sequence. Designed sequences and detailed results of the two-target pseudoknotted RNAs are tabulated in Additional file 1: Tables S4 and S5.

In addition to the randomly generated datasets, we constructed a two-target pseudoknot dataset based on natural RNA pseudoknots taken from Pseudobase [36]. The two-target ‘Pseudobase’ dataset (we call LE80 dataset) was constructed as follows: Step 1) we set *i* = 1; Step 2) we pick up the *i*-th pseudoknot from the Pseudobase dataset for single-target design [32, 36]; Step 3) we compare the *i*-th pseudoknot with *j*-th pseudoknot (*i*<*j*) one by one; if the *i*-th and *j*-th pseudoknots have the same nucleotide length and have a structure similarity = ([target structure length] - [the Hamming distance between the *i*-th and *j*-th pseudoknots in bracket notation])/(target structure length) < 0.8, we add a set of the *i*-th and *j*-th pseudoknots to the LE80 dataset and mark the *i*-th and *j*-th pseudoknots in the Pseudobase dataset for single-target design (marked pseudoknots are not used at a subsequent processing, therefore each pseudoknot in the Pseudobase dataset for single-target design can appear only once in the LE80 dataset); Step 4) we increment *i* by one; if we reach the last pseudoknot, stop the processing; otherwise go to Step 2. It is noted that, in the procedure from Step 1 to Step 4, we consider the pseudoknots with a length of ≥ 40 and ≤ 80 nucleotides.

After removing the target sets having a pseudoknot which cannot be dealt with by NUPACK, we finally obtained 34 target sets of two target pseudoknots as the LE80 dataset. Design results for the LE80 dataset are tabulated in Table 3 (desinged sequences and the other data can be seen in Additional file 1: Table S6). By using MODENA, we obtained 22 sets (65 % of the 34 sets) in which at least one target has the lowest free energy, and obtained only four sets (12 % of the 34 sets) having a completely multistable RNA sequence. Compared with the corresponding percentages of the PK80 dataset (90 % and 50 %, respectively), these results imply that designing multistable RNA sequences which have naturally occurring pseudoknots is more difficult than the RNA designs with the target pseudoknots generated by NUPACK:subopt.

**Table 3:** The design results for the two-target Pseudobase dataset (the LE80 dataset)

Pseudobase ID	*l*	*δ* *e*_1_	*δ* *e*_2_	*n*_1_	*n*_2_
PKB00002_PKB00004	50	0.20	0.20	5	0
PKB00005_PKB00015	41	0.50	0.50	6	0
PKB00008_PKB00031	40	0.00	0.70	6	0
PKB00010_PKB00066	40	0.00	0.00	22	3
PKB00012_PKB00268	40	0.00	0.20	13	0
PKB00030_PKB00045	41	0.20	0.90	9	0
PKB00047_PKB00069	61	4.00	4.40	0	0
PKB00048_PKB00265	61	0.50	1.20	0	0
PKB00050_PKB00128	59	0.00	0.00	12	1
PKB00052_PKB00107	52	0.00	0.10	7	0
PKB00057_PKB00072	67	3.60	3.90	0	0
PKB00068_PKB00129	68	4.80	4.90	0	0
PKB00070_PKB00244	55	0.00	0.50	3	0
PKB00078_PKB00106	62	0.00	0.40	8	0
PKB00080_PKB00132	49	0.20	0.20	13	0
PKB00088_PKB00127	62	0.20	0.30	2	0
PKB00098_PKB00232	62	0.80	1.40	0	0
PKB00131_PKB00205	48	0.00	0.01	29	0
PKB00139_PKB00141	70	1.30	1.90	0	0
PKB00142_PKB00231	71	0.10	0.60	0	0
PKB00143_PKB00233	71	2.50	2.60	0	0
PKB00148_PKB00218	72	3.90	4.90	0	0
PKB00175_PKB00259	57	1.60	1.60	3	0
PKB00179_PKB00280	68	1.50	1.70	3	0
PKB00180_PKB00212	64	0.10	0.30	4	0
PKB00190_PKB00266	47	0.00	0.00	29	7
PKB00207_PKB00213	45	0.00	0.00	12	2
PKB00211_PKB00239	80	0.30	0.40	3	0
PKB00222_PKB00305	80	2.10	3.20	1	0
PKB00224_PKB00281	43	0.00	0.10	10	0
PKB00230_PKB00273	48	0.00	0.40	7	0
PKB00248_PKB00257	66	0.40	2.10	0	0
PKB00263_PKB00270	62	0.20	0.60	0	0
PKB00269_PKB00272	66	1.40	1.40	0	0
mean	-	0.89	1.22	-	-
median	-	0.20	0.55	-	-

The explanations for *l*, *δ* *e*_1_, *δ* *e*_2_, *n*_1_, and *n*_2_ are the same as those of Table 2. The values of *δ* *e*_1_ and *δ* *e*_2_ are those for the designed sequence with the lowest *δ* *e*_2_ in the 30 designed sequences

### Three- and four-target designs

To evaluate the design performance for more than two target structures, we performed three- and four-target RNA sequence designs with 100 sets of target structures generated by applying RNAshapes [37] to the random RNA sequences which were used to generate the benchmark dataset in the paper of RNAdesign [26]. Here, we used *f*_1_, *f*_2_, and *f*_3_ as the objective functions. The results for the three- and four-target designs are tabulated in Table 4. As shown in Table 4, the RNA sequences designed by MODENA have the lowest means (0.27 kcal/mol for 3 str and 0.84 kcal/mol for 4 str) and medians (0.00 kcal/mol for 3 str and 0.39 kcal/mol for 4 str) of *δ* *e*_1_ among the three design algorithms. These results indicate that, compared with the other two design methods, MODENA successfully designed RNA sequences whose lowest energy target structure has the free energy very close to that of the ground state. In the case of the highest energy target structure (*δ* *e*_2_) of three-target designs, the RNA sequences designed by MODENA give a mean (0.54) and median (0.30) better than those (mean = 0.96 and median = 0.80) of Frnakenstein and comparable to those (mean = 0.53 and median = 0.30) of RNAdesign. In the case of the *δ* *e*_2_ of four-target designs also, MODENA (mean = 1.78 and median = 1.40) outperformed RNAdesign and Frnakenstein in terms of the mean and median. Detailed data of these designs are shown in Additional file 1: Tables S2 and S3.

**Table 4:** The results of three- and four-target designs

	MODENA		RNAdesign		Frnakenstein	
	3str	4str	3str	4str	3str	4str
mean (*δ* *e*_1_)	0.27	0.84	0.35	1.63^a^	0.39	0.92
median (*δ* *e*_1_)	0.00	0.39	0.05	0.70^a^	0.10	0.55
mean (*δ* *e*_2_)	0.54	1.78	0.53	2.31^a^	0.96	1.89
median (*δ* *e*_2_)	0.30	1.40	0.30	1.50^a^	0.80	1.60

The 3str and 4str indicate the results for the 100 target sets of three- and four-target designs, respectively. The definitions of the energy differences *δ* *e*_1_ and *δ* *e*_2_ in kcal/mol are described in Table 2

^a^Since the computational times for five target sets in the four-target designs were very long, we terminated the five runs of the 100 runs (a single run corresponds to one target set). For this reason, we calculated these means and medians of RNAdesign without the results of the five target sets

#### Multi-target RNA design with sequence constraints

To test the design performance with sequence constraints, we re-performed the RNA sequence designs with the sets of the two, three, and four pseudoknot-free targets, where a randomly selected subsequence with a length of 10 % of the original sequence is used as sequence constraints in each design problem. As a result, for the two-target designs, we obtained higher means and medians than those of the designs without the constraints, while results comparable to the designs without the constraints were obtained for the three- and four-target designs. Data of these designs are included in Additional file 1: Tables S7 - S9.

#### Single-target design

Since the multi-target version of MODENA can also perform single-target RNA design, the results for single-target designs without sequence constraints are also included in Additional file 2: Tables S10 – S15, where the results obtained for the RNAiFold datasets (including the EteRNA dataset) [20, 38] and the Pseudobase dataset [32, 36] are shown. Input files, in which the objective functions and the *methods* used for each single-target design are described, are available from the MODENA website.

### An example of RNA device design

As an example of RNA device design, we re-designed the ribozyme-based RNA device proposed by Win and Smolke [39]. This RNA device, as described in [15] in detail, utilizes the sequence motifs of sTRSV hammerhead ribozyme and theophylline aptamer to realize an ON switch which can be used by embedding it in the 3’-UTR of a mRNA. When a ligand does not exist, this ON switch folds into the ribozyme structure and catalyzes self-cleavage, leading to the low expression of the gene in the mRNA. This design problem becomes a two-target RNA design since ribozyme-active and -inactive conformations specify the function. This RNA device design has the following features: (i) The ligand-binding state of the RNA device is approximately modelled as the minimum free energy (MFE) structure predicted with the secondary structure constraints of the aptamer domain. Such a modelling is realized by utilizing the structure constraint function provided by a secondary structure prediction method. (ii) The target structure of the ribozyme-inactive state is specified by the loop nucleotides which disrupt the stem II of the ribozyme. (iii) Some target secondary structures (other than the ribozyme structure, the aptamer domain structure, and the loop disrupting the stem II) are specified by a wild card; the secondary structures of such regions are automatically determined during the design computation. As objective functions, we minimized *F*_1_=*E*(*Θ*_active_)−*G*, *F*_2_=|*E*(*Θ*_inactive_)−*E*(*Θ*_active_)−1.0|, *F*_3_=−(*σ*_inactive_+*σ*_active_)/2, *F*_4_=|*r*(GC:CONT)−50|, where *Θ*_inactive_ and *Θ*_active_ indicate the MFE structures of the inactive and active state, respectively; *σ*_inactive_ and *σ*_active_ are the structure similarities with the inactive- and active-target structures, respectively. RNAstructure 5.3 package was used to evaluate these objective functions. We ran MODENA to design the RNA device mentioned above with option “-conv 0”, which guarantees that the run does not stop until the specified maximum number of generations (in this case, 200) is reached.

**Fig. 8:**
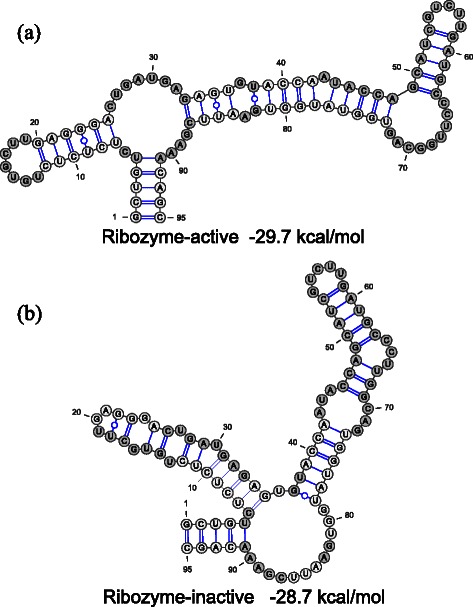
A design example of ribozyme-based RNA device. Gray circles indicate sequence constraints. The original ribozyme consensus sequence motifs and loop nucleotides of L2bulge1 [15] were used as sequence constraints. The structures and free energies shown in this figure were computed by Fold in RNAstructure package. **a** Ribozyme-active conformation predicted as the MFE structure. **b** Ribozyme-inactive conformation predicted as the MFE structure when the aptamer domain structure is constrained; the region ranging from 43 to 72 nt is the theophylline aptamer domain. The designed sequence is GCUGUCUCUCUCUGUGCUUGAGGGACUGAUGAGAGUGUACCAAUACCAGCAUCGUCUUGAUGCCCUUGGCAGU GGUAUGGUGAAUUCGAAACAGC. These structures were visualized by VARNA [40]

As the design result, we obtained one RNA sequence which folds into a ribozyme-active conformation as the ground state; moreover, when the aptamer secondary structure is fixed by the structure constraints, this designed RNA folds into the secondary structure in which the stem II of the ribozyme is missing (these two structural features correspond to *F*_3_ = -1.0). The predicted structures of the designed sequences are shown in Fig. 8. In addition, this designed RNA has an energy difference of 1.0 kcal/mol between the active and inactive conformations (this corresponds to *F*_2_ = 0.0). This energy difference is exactly the same as the specified value in objective function *F*_2_. This design result indicates that MODENA is useful for designing RNA devices with complex features.

Since the energy barrier height between target structures can be one of the important features of RNA device, in addition to the RNA device design mentioned above, we designed RNA sequences by minimizing five OFs, i.e. *F*_1_, *F*_2_, *F*_3_, *F*_4_ and the energy barrier height *F*_5_ between two secondary structures, where the two secondary structures are the MFE structures predicted by RNAfold with and without the structure constraints for the aptamer domain. In this test design, we minimized *F*_5_; this can increase the transition probabilities between ribozyme-active and -inactive conformations when the ligand does not exist. We used the Vienna RNA package instead of RNAstructure package in this RNA design taking energy barrier height into account; this is because the barrier height prediction program, accessFindPath.py [34] called in MODENA with a look-ahead parameter of 1000, utilizes findpath.c [24] which is taken from the Vienna RNA package. We used the GA parameter settings similar to the RNA device design mentioned above except for a population size of 200 and the maximum number of generations of 400. By using MODENA, we successfully obtained two designed RNA sequences which have *F*_2_ = 0.0 and *F*_3_ = -1.0. Both of these designed RNA sequences have a predicted energy barrier height of 5.2 kcal/mol, which is a much smaller value than that (11.9 kcal/mol) predicted for the RNA sequence (shown in Fig. 8) designed by not taking energy barrier height into account. The designed sequences and their structures are shown in Additional file 3: Figure S2. It is noted that the predicted barrier height only gives an upper limit of the lowest barrier height since the algorithm of findpath.c is a heuristic algorithm.

### Computational time

The computational time of MODENA mainly depends on GA population size, convergence criterion, the maximum number of generations, the number of OFs, the size of a connected component, the length of target structures, and the *method* used to evaluate the OFs of the design. Mean computational times of the designs performed in the present study are tabulated in Table 5. It is noted that these designs utilize a simple parallelization based on OpenMP. If the user runs MODENA without the parallelization option, the computational times will become longer. As can be seen from Table 5, the RNA device designs and two-target pseudoknot designs took long computational times. These are due to the long computational times of the *methods* used in these designs. Faster structure prediction methods will be necessary to accelerate these designs. Even in the case of pseudoknot-free designs, we recommend users to limit the sequence length to a few hundreds of nucleotides at most to obtain good design results within a reasonable computational time. In Table 5, computational times of Frnakenstein and RNAdesign are also shown. As can be seen from the ‘two targets’ row, MODENA needed a much smaller mean computational time for the two-target designs compared with Frnakenstein.

**Table 5:** Computational times

		MODENA				
Dataset	*l* (nt)	pop. size	#threads	*t*_MODENA_ (s)	*t*_Frnakenstein_ (s)	*t*_RNAdesign_ (s)
two targets	149	100	4	446^a^	13,988^a^	2,924^c^
three targets	100	100	4	339^a^	1,924^b^	1,249^c^
four targets	100	100	4	385^a^	2,848^b^	1,653^c^
RNA device^d^	95	100	4	2,428^a^	-	-
RNA device^e^	95	200	6	15,460^b^	-	-
PK60	60	30	3	1,997^b^	-	-
PK80	80	30	3	7,780^b^	-	-
LE80	61	30	3	2,140^b^	-	-

An *l* indicate the mean length for each dataset. The ‘pop. size’, ‘#threads’, and *t*_MODENA_ columns indicate GA population size, the number of OpenMP threads used to parallelize a design, and the mean computational time of MODENA for each dataset, respectively. For example, from the ‘two targets’ row of this table, we can see that one MODENA run of the two-target design with a population size of 100 and four OpenMP threads took 446 seconds on average. PK60, PK80, and LE80 are the two-target datasets with pseudoknot. The computational times for “frnakenstein.py -s 100” and RNAdesign with option “-n 500 –thin 200 -b 100 –scale 1” are shown in *t*_Frnakenstein_ and *t*_RNAdesign_ columns, respectively

^a^Computational times measured on a PC with Intel Xeon E5-2603 (1.80 GHz) and 16 GB of memory (CentOS 5.9)

^b^The times measured on a PC with Intel Xeon E5-2665 (2.40 GHz) and 132 GB of memory (CentOS 6.4)

^c^Computational times measured on a PC with Intel Core i3-2100 (3.10 GHz) and 3.6 GB of memory (Fedora 18, which was installed in order to execute RNAdesign)

^d^and ^e^ indicate RNA device designs which take and does not take energy barrier height into account, respectively

^e^uses the maximum number of GA generations of 400

In the present study, we did not tune the GA parameters. The parameter values used in the present study were determined based on our past experience in the GA studies including RNA sequence alignment [31] and single-target RNA inverse folding [28, 32]. Some parameter tuning could improve the benchmark results shown in the present paper.

### Effect of the crossover for multiple targets

To examine the effect of the crossover for multiple targets, we performed the two-target designs of the 17 RNAtabupath target sets without the crossover for multiple targets (option “-opCr 0” was used for the purpose). Aa a result, we obtained the means for *δ* *e*_1_ and *δ* *e*_2_ (0.46 and 0.85, respectively), which are slightly worse values compared with the designs with the crossover operator (as can be seen in Table 2, 0.38 and 0.76, respectively). When we designed without the crossover operator, the medians for *δ* *e*_1_ and *δ* *e*_2_ were 0.1 and 0.3, respectively. The median of *δ* *e*_2_ is slightly better than that of the designs with the crossover operator (0.5). Wilcoxon’s signed rank tests for these comparisons (*P* = 0.9097 and 0.3296) indicate that the RNA sequences designed with and without the crossover are comparable with respect to *δ* *e*_1_ and *δ* *e*_2_. In Additional file 3: Figure S3, the Boltzmann probabilities of the RNA sequences designed with and without the crossover operator are shown. As can be seen from the figure, MODENA with the crossover designed the RNA sequences with the Boltzmann probabilities larger than MODENA without the crossover in many target sets; it is noteworthy that the designs with the crossover gave larger probabilities compared to those without the crossover in all the target sets with long lengths (> 200 nucleotides; HIV-1 leader, rb5, ribD leader, and s-box leader), while both are comparable for short sequences (< 100 nucleotides; dsrA, ms2, s15, and SL).

### Differences between the multi-target and previous versions of MODENA

Main differences between the new multi-target version and the previous single-target versions are as follows: (i) The previous versions cannot perform the RNA inverse folding with multiple target structures. The previous versions can accept only one target structure as an input. To perform the RNA inverse folding with multiple target structures, it is necessary to take the dependency graph into account in the initialization, mutation, and crossover as implemented in the multi-target version. (ii) The error diagnosis operator of the previous versions has been replaced by negative and positive mutation operators in the multi-target version. (iii) The ‘Mutation of undesired sequence motifs’ operator has been introduced in the new version. (iv) Objective functions more than two can be used in the multi-target version. More sequence *properties* including a predicted energy barrier height and a GC content can be used in the OFs in the multi-target version. In addition, not only sequence *property* values themselves, but also a function of sequence *property* values can be utilized in an objective function in the new version. The previous versions of MODENA can use only the two objective functions (a stability score and structure similarity score). (v) The secondary structure constraint, which is useful for modelling the ligand-binding state of an aptamer, has been introduced in the new version. (vi) The new version explores Pareto optimal solutions in the default setting, while the previous versions do weak Pareto optimal solutions. (vii) A simple parallelization of the evaluation part in the GA has been introduced in the new version.

### Differences between genetic algorithms (MODENA and Frnakenstein)

Important differences between MODENA and Frnakenstein are as follows. First, MODENA adopts a multi-objective genetic algorithm which can obtain the approximate Pareto optimal solutions at one run, while Frnakenstein designs RNA sequences based on the approach utilizing a weighted sum of objective functions (which includes the case of a single objective function) which explores one of the Pareto optimal solutions as the single optimal solution. MODENA uses multiple OFs such as *f*_1_, *f*_2_, and *f*_3_, whereas Frnakenstein uses a single OF as the default setting for multi-target RNA design. Due to this algorithmic difference, the stopping criterion (the number of continuous GA generations which have the same number of non-dominated solutions) of MODENA is also different from that of Frnakenstein.

Secondly, in the default setting of Frnakenstein, RNA sequences are initialized by RNAinverse where a target structure is randomly selected from multiple targets. This initialization does not guarantee the compatibility with multiple target structures. MODENA initializes RNA sequences with the nucleotide assignment algorithm which guarantees the compatibility.

Thirdly, MODENA has various functions which are useful for practical RNA device design and are not implemented in Frnakenstein: sequence and structure constraints, pseudoknot design function, target GC content, mutation of undesired sequence motifs, and OpenMP parallelization. Although, in principle, it may be possible to add these functions to Frnakenstein, such a version of Frnakenstein is currently not available.

Fourthly, MODENA is written in C and partially in C++ and Frnakenstein is a python program.

Finally, MODENA uses originally-developed GA operators different from those of Frnakenstein. As the default setting, the GA operators of Frnakenstein use base pairing probabilities, whereas the GA operators of MODENA do not utilize base paring probabilities. RNA folding with base paring probability computation takes a longer time than that without base paring probabilities, this difference could be one of the reasons for the difference between the computational times of MODENA and Frnakenstein.

### Average pairwise structure distance of target structures

To know how diverse structures are included in each set of target structures, we calculated the average pairwise structure distance (APSD) for each set of target structures. The calculated APSD is displayed in the filename of each set of target structures, e.g. ‘alpha_operon-apsd13.inp’ indicates a set of target structures with an APSD of 0.13. The target structures (input files) used in the benchmarks can be downloaded from the MODENA website.

### Availability

Academic users can use MODENA from our website (http://rna.eit.hirosaki-u.ac.jp/modena/multi).

## Conclusion

We have presented a novel multi-objective genetic algorithm useful for multi-target RNA design. To enable us to design the RNA sequences in which multiple target structures have the free energies close to that of the ground state, we developed novel initialization, mutation, and crossover procedures which take the dependency graph into account. We examined the design performance of the present algorithm with the various sets of two, three, and four target structures and obtained good design performances compared with the other state-of-the-art multi-target RNA design algorithms. As an example of the practical RNA device design with sequence and structure constraints, we presented successfully designed RNA sequences which have the characteristic sequence and structural features specifying the function of a ribozyme-based RNA device. Although we showed only one example of RNA device design, MODENA can design RNA devices responding to various ligands by changing the secondary structure and sequence motif of the aptamer domain. Development of design templates (input files for MODENA) for various RNA devices, including RNA devices requiring four target structures (e.g. AND and OR logic gates [2, 11]), is currently in progress.

By virtue of the modular nature of genetic algorithm in which a solution evaluation part and the other optimization parts are clearly separated as different algorithmic parts, MODENA can perform multi-target RNA sequence design based on various widely-used RNA secondary structure prediction methods. It is noteworthy that we can use MODENA to design multistable pseudoknots, whereas the previous inverse folding methods cannot design multistable pseudoknots.

The current version of MODENA has several issues to be improved. For example, addition of RNA-RNA interaction prediction algorithms to the *methods* invoked in MODENA will make MODENA possible to design interacting structured RNA sequences. Such a design tool is useful for automatic design of RNA-RNA interaction circuits [3]. Since the computational speed of the current version is not so fast and the parallelization is very simple, more sophisticated parallelization will improve the speed of MODENA. Computational speed is important not only for standalone use, but also for the webserver for designing RNA devices.

From our experience, we can say that reducing the number of GU base pairs is important to obtain a good design result. For this reason, avoiding a subset of the sequence space having many GU base pairs is desirable for RNA sequence design. Since our sequence sampling approach, the nucleotide assignment algorithm, is rather empirical, development of a more sophisticated GU base pair sampling method is an interesting direction for future research. It is noteworthy that, by introducing more random nature into the nucleotide assignment algorithm, we can give non-zero sampling probabilities to all compatible nucleotide assignments (some weights will be needed to reduce the number of GU pairs).

It is noted that the present algorithm does not guarantee the desired *in vitro*/*in vivo* function of the designed RNA sequences. The functions of designed RNAs have to be determined by experiment. We hope that our multi-objective optimization approach gives an effective guide for developing novel RNA devices which work *in vitro*/*in vivo* and leads to fruitful collaboration between informatics and experiment in the field of biomolecular device design.

## Additional files

Additional file 1
**Supplementary Tables S1 – S9.** The detailed results of the RNA sequences designed by MODENA. The results for two-, three-, four-target designs with and without sequence constraints are included. (XLSX 71.7 kb)

Additional file 2
**Supplementary Tables S10 – S15.** The detailed results of the single-target RNA design by MODENA. (XLSX 70.8 kb)

Additional file 3
**Supplementary Figures S1 – S3.** **Figure S1.** Boltzmann probabilities of the designed RNA sequences by MODENA, Frnakenstein, and RNAdesign for the two-target RNAtabupath dataset. **Figure S2.** An example of the ribozyme-based RNA device design which takes energy barrier height into account. **Figure S3.** Boltzmann probabilities of the RNA sequences designed by MODENA with and without the crossover operator for the two-target RNAtabupath dataset. (PDF 557 kb)
