# Supplementary material for: Multi-objective optimization for RNA design with multiple target secondary structures
Source: BMC Bioinformatics. 2015 Sep 3;16:280. doi: 10.1186/s12859-015-0706-x (PMC4559319; doi:10.1186/s12859-015-0706-x)
Supplement: Additional file 3 — Supplementary Figures S1 – S3. Figure S1. Boltzmann probabilities of the designed RNA sequences by MODENA, Frnakenstein, and RNAdesign for the two-target RNAtabupath dataset. Figure S2. An example of the ribozyme-based RNA device design which takes energy barrier height into account. Figure S3. Boltzmann probabilities of the RNA sequences designed by MODENA with and without the crossover operator for the two-target RNAtabupath dataset. (PDF 557 kb) [file 12859_2015_706_MOESM3_ESM.pdf]

Additional file 3:
Multi-objective optimization for RNA design with multiple target secondary structures
A. Taneda

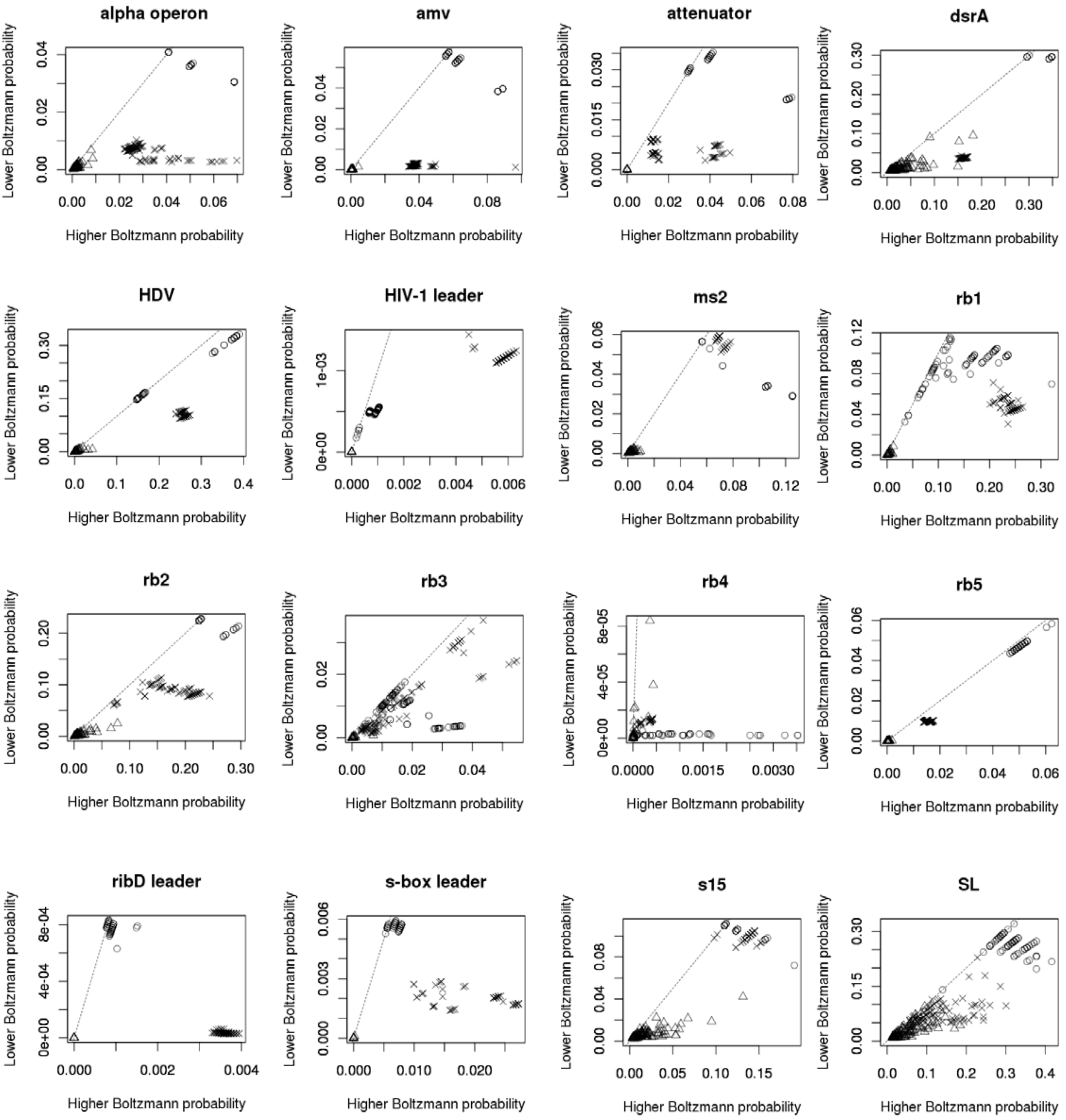

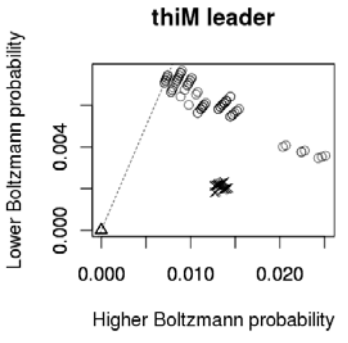

Additional file 3:Figure S1 Boltzmann probabilities of the designed RNA sequences by MODENA (denoted by circles), Frnakenstein (denoted by crosses), and RNAdesign (denoted by triangles). The results for 17 target secondary structure sets of the RNAtabupath dataset are shown. “Higher Boltzmann probability” and “Lower Boltzmann probability” indicate the probability of the target structure with a higher probability and that with a lower probability, respectively. Each method has 100 designed RNA sequences in each target secondary structure set. A dashed line indicates $y = x$ in each plot; a marker plotted on a dashed line corresponds to a designed sequence in which two target structures have the same Boltzmann probability.

(a)

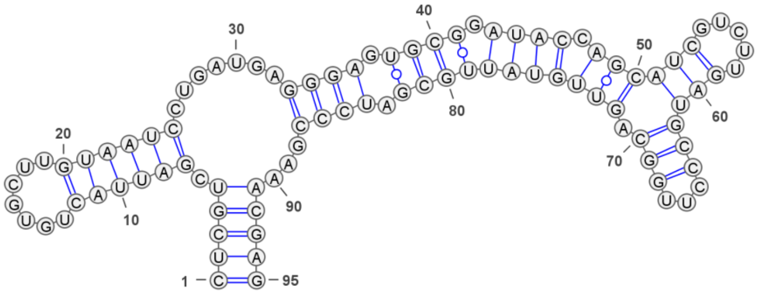

Ribozyme-active -27.9/kcal/mol

(b)

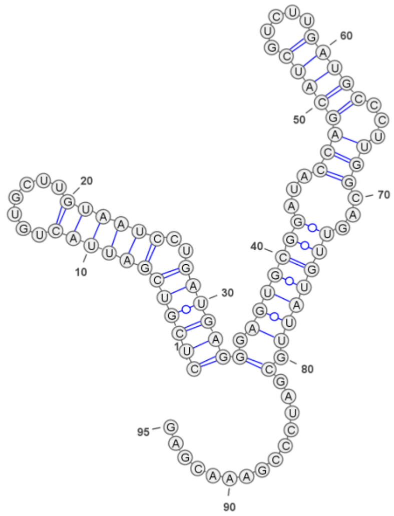

Ribozyme-inactive -26.9/kcal

Additional file 3:Figure S2 An example of the ribozyme-based RNA device design which takes energy barrier height into account. Sequence constraints shown in Figure 5 were used. The structures and free energies shown in this figure were computed by RNAfold with option –d2. (a) Ribozyme-active conformation predicted as the MFE structure. (b) Ribozyme-inactive conformation. In addition to the RNA sequence (CUCGUCGAUUACUGUGCUUGUAAUCCUGAUGAGGGAGUGCGGAUACC
AGCAUCGUCUUGAUGCCCUUGGCAGUUGUAUUGCGAUCCCGAAACGAG) shown in this figure, we obtained another designed sequence (CUCGUCGCACGCUGUGCUUGUGUGCCUGAUGAGGGAGUGCGGAUACCA
GCAUCGUCUUGAUGCCCUUGGCAGUUGUAUUGCGAUCCCGAAACGAG) which folds into exactly the same ribozyme-active (-31.6 kcal/mol) and –inactive (-30.6 kcal/mol) structures as those of (a) and (b), respectively. These structures were visualized by VARNA.

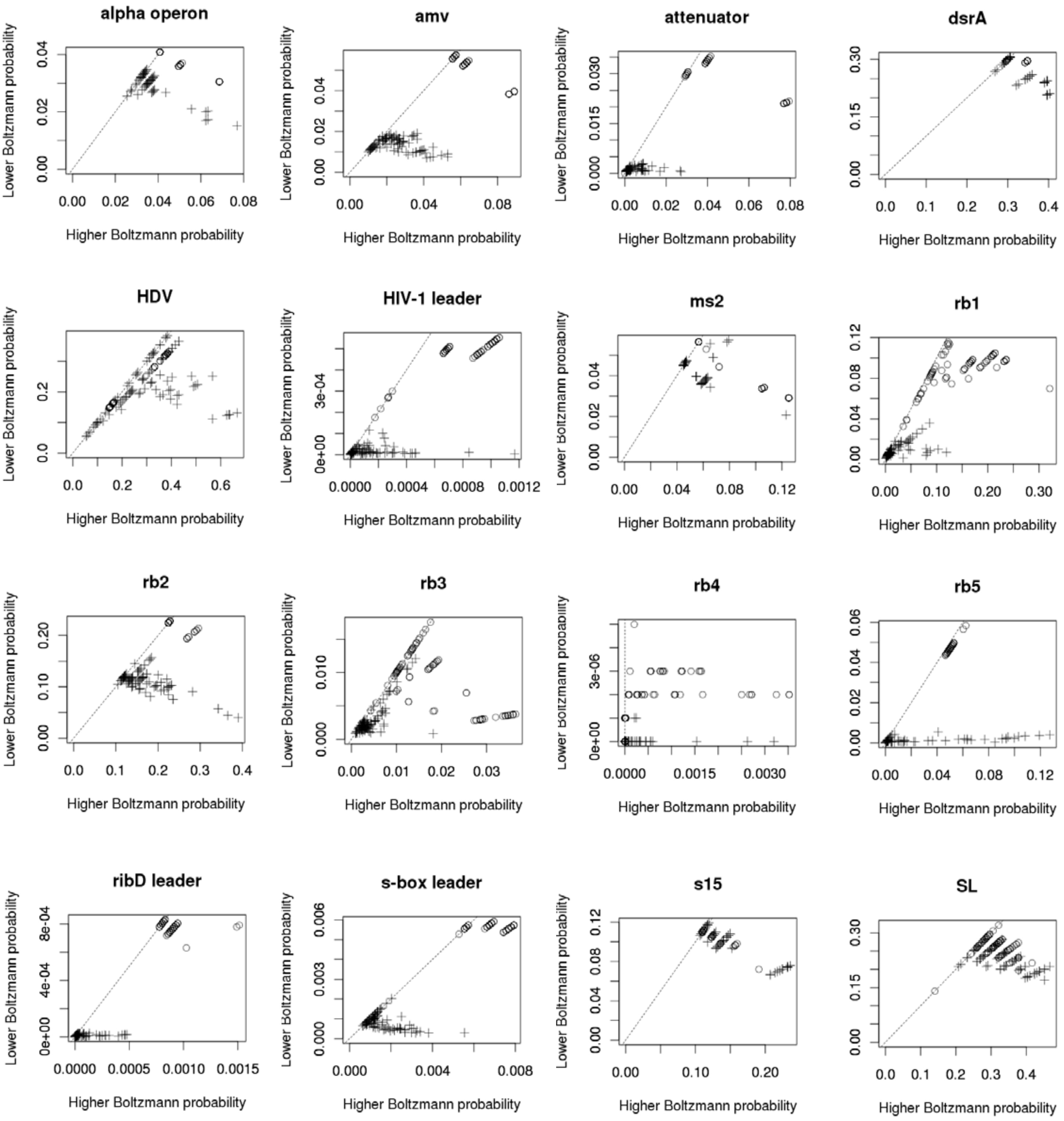

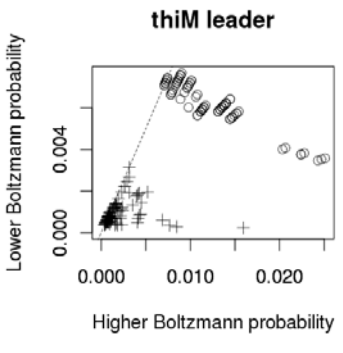

Additional file 3:Figure S3 Boltzmann probabilities of the designed RNA sequences by MODENA with the crossover for multiple targets (denoted by circles) and MODENA without the crossover (denoted by +). The results for 17 target secondary structure sets of the RNAtabupath dataset are shown. The other explanation is the same with the caption of Additional file 3:Figure S1.
